# Size distributions and dispersions along a 485-year chronosequence for sand dune vegetation

**DOI:** 10.1002/ece3.62

**Published:** 2012-04

**Authors:** Jennifer M Waugh, Lonnie W Aarssen

**Affiliations:** Department of Biology, Queen's UniversityKingston, ON, K7L 3N6, Canada

**Keywords:** Coexistence, competition, competitive ability, plant size, seed size, succession

## Abstract

Using a sand dune chronosequence that spans 485 years of primary succession, we collected nearest-neighbor vegetation data to test two predictions associated with the traditional “size-advantage” hypothesis for plant competitive ability: (1) the relative representation of larger species should increase in later stages of succession; and (2) resident species that are near neighbors should, over successional time, become more similar in plant body size and/or seed size than expected by random assembly. The first prediction was supported over the time period between mid to later succession, but the second prediction was not; that is, there was no temporal pattern across the chronosequence indicating that either larger resident species, or larger seeded resident species, increasingly exclude smaller ones from local neighborhoods over time. Rather, neighboring species were generally more different from each other in seed sizes than expected by random assembly. As larger species accumulate over time, some relatively small species are lost from later stages of succession, but species size distributions nevertheless remain strongly right-skewed—even in late succession—and species of disparate sizes are just as likely as in early succession to coexist as immediate neighbors. This local-scale coexistence of disparate sized neighbors might be accounted for—as in traditional interpretations—in terms of species differences in “physical-space-niches” (e.g., involving different rooting depths), combined with possible facilitation effects. We propose, however, that this coexistence may also occur because competitive ability involves more than just a size advantage, with traits associated with survival (tolerance of intense competition) and fecundity (offspring production despite intense competition) being at least equally important.

## Introduction

Competitive ability in plants has been linked traditionally to both relatively large plant size and relatively large seed size (Gaudet and Keddy 1988; Grace and Tilman 1990; Weiner 1990; Goldberg 1996). Larger plants are often viewed as superior competitors because competition is typically asymmetric, at least for light (Schwinning and Weiner 1998). Large seed size also appears to affect growth rate and survival of seedlings under competition for a variety of species (Leishman et al. 2000). If competition is intense and relative size is the principal determinant of competitive ability in plants then this should be evident from an overdispersion pattern—that is, species with small- and large-sized traits should be found in proximity less often than expected by chance (e.g., Epp and Aarssen 1989; Weiher et al. 1998; Hubbell et al. 2001; Schurr et al. 2004; Stubbs and Wilson 2004; Turnbull et al. 2004; Schamp et al. 2008).

Few previous studies of trait dispersion have spanned more than a few years. The sand dune chronosequence used in the present study, however, allowed an assessment of trait dispersion patterns during plant community assembly across a time span of several centuries. This allowed us to search for changing patterns of trait dispersion over time as potential indicators of the increasing importance of competition through succession. As tests of the “size-advantage” hypothesis for competitive ability, we addressed two predictions: (1) larger species should, over time, become more abundant relative to smaller species, reflected by size distributions that become increasingly less right-skewed over time; (2) over successional time, there should be increasing evidence that resident species that are near neighbors are more similar in plant size and/or seed size than expected by random assembly (reflecting the prediction that larger species or larger seeded species should exclude smaller ones from local neighborhoods).

**Figure 1:**
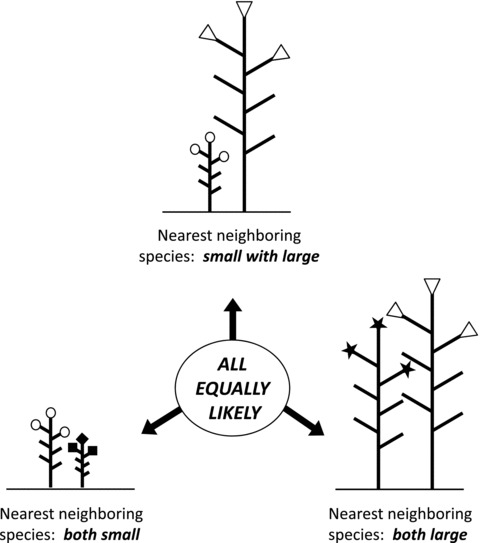
Pictorial illustration of the null hypothesis for the present study: nearest-neighboring pairs of species are generally not different from each other in their relative body sizes than expected from a random pair-wise assembly of resident species.

The spatial pattern that is implied by the size-advantage hypothesis is that small species/individuals will become increasingly segregated through time from large species/individuals at a spatial scale equal to or larger than the interaction between neighboring individuals. The corresponding null hypothesis is that the relative sizes of neighboring individuals will be random throughout the dune chronosequence (Fig. 1).

## Materials and Methods

### Study site

The study was conducted on a series of dune-capped beach ridges located in Wilderness State Park in Emmet County of northern Lower Michigan, United States (45°43′N, 84°56′E). Because of its park status, most of the study site appeared to have been protected from the impact of major human disturbance (pers. obs. J. M. Waugh). Dating by Lichter (1995, 1997) indicates that the 108 dunes represent a chronosequence spanning approximately 3500 years, and that new dunes are formed every 32 years (on average) for the 72 most recent dunes. The focus in this study is on the first 17 dunes in the northern region of the dune chronosequence, which fully covers the progress through approximately 485 years of primary succession, with very young dunes dominated by marram grass (*Ammophila breviligulata* Fernald), and late stages of succession dominated by mature red pine forest (*Pinus resinosa* Aiton). A highly significant linear relationship exists between estimated dune age based on data from Lichter (1995, 1997, 1998) and dune number (*r*^2^= 0.99, *n*= 17, *P*≪ 0.001). Consequently, dune number is used as a proxy for community age in our analyses.

Lichter (1998) reported that soil moisture, soil nitrogen, and soil phosphorus increase across the dune chronosequence, and total carbon and nitrogen in the upper 15 cm of mineral soil reach steady-state levels by dune 6. Wind velocity on the dunes, and the degree of evaporation and soil drying are related to whether the dunes are open or forested sites. In this study, the forest species (i.e., tree species such as *Thuja occidentalis*, *Abies balsamea*, *Picea glauca*) first appear on dune 5. Lichter (1998) quantified sand aggregation on early dunes, which limits seed germination for some species (Weller 1989; Zhang and Maun 1990), and burial of established seedlings, which may cause mortality if growth is outpaced by the rate of sand deposition (Maun and Lapierre 1984; Harris and Davy 1987). The first four dunes are actively accreting sand (0.1–3 cm/year on dunes 2–4), while the fifth and sixth dune have roughly zero net accumulation (Lichter 1998) and can therefore be considered stable environments. Larger, longer lived species were first sampled in this study on dune 5, since this is the first stable dune with respect to sand disturbance. Additional details of vegetation composition and structure are reported in Waugh and Aarssen (2011).

### Vegetation sampling

Vegetation surveys were conducted between mid-June and late August 2004. A linear transect running the length of the dune (i.e., parallel to the shore) was established on each dune. Because dunes varied in width and height, the transect was run along the center of each dune at the dune apex. With the exception of dune 3, a 1500-m transect was delineated on dunes 1 through 10. Sampling on dune 3 was truncated to a 500-m transect because the dune only began in the southern third of the selected study region of the dune chronosequence. The remaining dunes (dunes 11 through 17) were sampled along a ∼1250-m transect; the southern portion of these older dunes had discontinuities (blowouts or parabolic dunes) that rendered definitive identification of the dunes impossible, and therefore created uncertainty about the age and historic stability of the dunes in that section. Thus, the sampled portion of the chronosequence was selected to obtain the largest continuous portion of the chronosequence that would allow for avoidance of the blowouts/parabolic dune sections previously identified by Lichter (1998).

The locations of sampling points were identified using a modified stratified random sampling method: the first sampling point on each dune was randomly selected in the first 8 m of the transect. The second sampling point was randomly selected within 3–8 m of the first point, and subsequent sampling points followed the pattern of random selection within 3–8 m of the previous point. The minimum distance of 3 m between sampling points was imposed in order to reduce the potential for spatial autocorrelation among samples, and to ensure that no individual plant was sampled twice. Sample sizes ranged from 49 (dune 3) to 169 points per dune (with a mean of 146 points). Sampling was conducted using a nearest-neighbor design. At each sampling point, the nearest rooted individual was identified as the “point” species. The conspecific individual that was rooted nearest to the point species was identified as the intraspecific, or “self” neighbor. Similarly, the closest neighboring individual belonging to a different species was identified as the interspecific or “non-self” neighbor. Individuals were defined as a rooted stem. For species capable of underground spreading (e.g., by rhizomes), no attempt was made to distinguish intraspecific individuals (ramets) that may have been connected belowground. The identification of nearest neighbors based on rooting proximity was adopted because it was assumed this would reflect the most appropriate scale for both above- and belowground resource competition.

For all point and neighboring individuals, the height was measured from ground to the highest point on the plant. For large trees, height was estimated using a clinometer (Suunto Precision Instruments, Finland). The distance (from stem to stem) was measured from the point species to its nearest self-neighbor (distance to self) and to its nearest nonself-neighbor (distance to nonself).

All vascular plants were included in the survey. Botanical nomenclature follows Gleason and Cronquist (1991). Species in the *Amelanchier* and *Viola* genera could not be positively identified to species level (due to lack of flowering/fruiting material) and thus were identified only to genus. Similarly, the identification of six species of plants could not be determined, but the individuals were consistently recognized as distinct species during sampling and were treated as separate code-named species during analysis.

### Size distribution analysis

Mean maximum species size for each dune was determined by compiling maximum height data for each species from Gleason and Cronquist (1991), and where necessary from, Soper and Heimburger (1994), Cody and Britton (1989), Legasy (1995), and Farrar (1995). For five unknown species for which a maximum height could not be obtained from literature, the maximum measured height across all dunes was used as an estimate. Skewness coefficients (Zar 1999) of the distribution of maximum species sizes for each dune were compared across all 17 dunes to assess whether there was a directional change in the skew of the distribution through succession.

### Trait dispersion analyses

To assess the extent to which species are spatially associated with species of similar (or dissimilar) size, the average size difference (ASD) between interspecific neighbors for each dune was calculated and compared to a distribution of possible ASDs. The distribution of ASDs was determined by creating pairs of heights (a measure of size), calculating the absolute difference in height for each pair, and then averaging the absolute height differences across all pairs for a given dune. Pairs were created by randomly choosing (without replacement) two heights from the collection of all point and neighbor species heights on a given dune. This complete randomization of heights effectively removed any potential influence that species abundance had on determining whether an individual (and therefore its associated height) was a point or neighbor species.

The ASD between neighbors on a dune was considered to be significantly smaller than expected by chance if the observed ASD fell into the bottom 2.5% (250 samples of 10,000), and significantly larger than expected if the observed ASD fell into the top 2.5% of the distribution of possible ASDs. Small absolute ASDs would be consistent with neighbors being of similar size, while large absolute ASDs would reflect neighbors of disparate sizes.

Absolute ASDs were calculated for nearest neighbors using measured size, and for interspecific neighbors using two separate size datasets: maximum recorded size, and measured size. “Maximum recorded size” refers to the maximum size data collected from Gleason and Cronquist (1991). “Measured size” is the actual measured height recorded for each individual sampled on a dune. For any given species on a dune, then, there may be as many distinct measured sizes as there are individuals of that species sampled.

A similar randomization was conducted using seed size. To assess the extent to which species are spatially associated with species having similar (or dissimilar) seed size, the absolute ASD between seed size of neighbors was calculated for each dune and compared to a distribution of possible absolute ASDs between seed sizes. The randomization procedure and interpretation are as described for the height randomizations above. Seed size data were compiled for 77 of the 95 identified species. For the majority of the 77 species, seed size data were compiled from Montgomery (1977); the remainder of the data was obtained from Gleason and Cronquist (1991), Mohlenbrock (1999), Legasy (1995), Kucera (1998), or Fernald (1960) where possible. Seed data were not obtained for 18 species, six of which were the unidentified species and four were ferns or *Equisetum* species (having spores rather than seeds).

The significance levels for each set of size randomizations (e.g., interspecific neighbors using measured size) were adjusted by sequential Bonferroni corrections (Quinn and Keough 2002) to account for the increased probability of a Type 1 error. All randomizations were performed in MATLAB 7 Student Version.

## Results

A total of 95 vascular plant species were identified as point and/or neighbor species across the dune chronosequence.

### Size distributions

**Figure 2:**
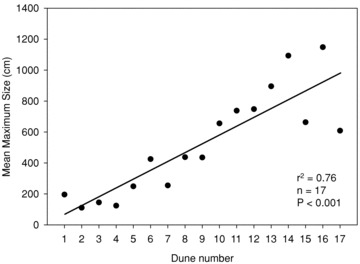
Linear regression analysis of mean maximum size (height) of species for a dune versus dune number. Mean maximum size is the mean of the maximum recorded sizes for species present on a dune, regardless of relative frequency of each species.

**Figure 3:**
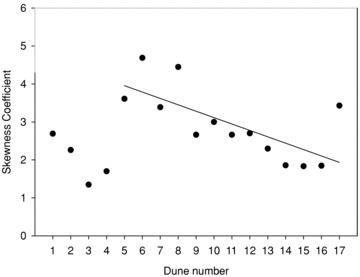
Relationship between the maximum size distribution skewness coefficient (Zar 1999) for each dune and dune number. A skewness value greater than zero indicates a distribution that is skewed to the right. That is, the dune has a relatively large number of relatively small species. The linear regression covers dunes 5–17 only because these dunes were considered to be stable environments (*r*^2^= 0.48, *n*= 13, *P*= 0.008).

Mean maximum size of species increased with dune number (*r*^2^= 0.76, *n*= 17, *P*≪ 0.001; Fig. 2). The skewness of the maximum size distribution of species on each dune showed no discernable temporal pattern over the entire chronosequence, but decreased (*r*^2^= 0.48, *n*= 13, *P*= 0.008) from dune 5 through 17 (Fig. 3).

### Trait dispersion

On seven dunes (1, 4, 5, 6, 13, 15, and 16), the maximum recorded absolute ASD between interspecific neighbors was significantly large, indicating that the point and neighbor species were on average more different in size than expected by chance (Table 1). After sequential Bonferroni corrections, the interpretation of the maximum recorded absolute ASD of dunes 13 and 16 became nonsignificant.

**Table 1:** Probabilities^1^ of observed average size difference (ASD) between interspecific or nearest neighbors based on various species size measures.

	Interspecific neighbors			Nearest neighbors
Dune Number (age; yr)	Maximum recorded size	Measured size of individuals	Published seed size	Measured size of individuals
1 (13)	**0.9999**	**0.9962**	**0.9993**	**0.0001**
2 (38)	0.2308	**1.0000**	**1.0000**	**0.0001**
3 (68)	0.9670	0.7054	**0.9999**	**0.0006**
4 (108)	**0.9971**	0.8637	**0.9997**	**0.0001**
5 (133)	**0.9999**	0.2096	0.9142	**0.0001**
6 (158)	**0.9973**	0.6820	0.9811*	**0.0001**
7 (188)	0.9293	0.0399	0.6755	**0.0001**
8 (238)	0.1591	0.0961	0.7765	**0.0001**
9 (298)	0.1749	0.0992	0.3322	0.0056*
10 (328)	0.9182	0.1785	0.9589	**0.0001**
11 (358)	0.5433	0.0232	0.9301	**0.0014**
12 (385)	0.7216	0.4669	0.6909	**0.0020**
13 (413)	0.9823*	0.5894	**1.0000**	0.0605
14 (433)	0.9627	0.5036	0.9965*	**0.0253**
15 (453)	**1.0000**	0.9826*	**1.0000**	0.0623
16 (478)	0.9906*	0.9478	**0.9996**	0.0640
17 (498)	0.7458	0.0783	0.8838	**0.0001**

1 Probabilities ≤0.0250 (in bold type) indicate ASDs that are smaller than expected by chance and probabilities ≥0.9750 (in bold type) indicate ASDs larger than expected by chance.

* Test result that is no longer significant after sequential Bonferroni correction.

The absolute ASD between interspecific neighbors (i.e., between point and nonself-neighbors) using measured sizes was significantly large for three dunes (1, 2, and 15). The absolute ASD on dune 11 was significantly small, indicating that the point and nearest-neighbor heights on dune 11 were, on average, more similar in size than expected by chance. After sequential Bonferroni corrections, the ASD was no longer significant on dune 15 (Table 1).

The absolute ASDs based on measured size between nearest neighbors were significantly smaller than expected by chance on 14 dunes; the absolute ASDs for the remaining dunes (13, 15, and 16) were nonsignificant. After sequential Bonferroni corrections, the ASD on dune 14 was no longer significantly small (Table 1).

The absolute ASDs between seed size of neighbors were significantly large for nine dunes (1, 2, 3, 4, 6, 13, 14, 15, and 16), indicating that the seed sizes of point and interspecific neighbors were, on average, more different than expected by chance. After sequential Bonferroni corrections, the absolute ASDs between seed size were no longer significantly large for dunes 6 and 14 (Table 1).

## Discussion

Young dunes have only relatively small species and older dunes host progressively larger species (Fig. 2). This trend is consistent with virtually every other known pattern of vegetation succession; initial colonists are relatively small and often shorter lived, and are typically replaced by, or excluded because of competition from, larger longer lived species that tend to establish later. The highest species richness then generally occurs in intermediate stages of succession where some of the earlier colonists still persist (Grime 1979), and this is also the case for our study site (Waugh and Aarssen 2011).

For the dune sequence as a whole, the relative number of large resident species did not increase consistently over time; that is, there was no consistent temporal pattern in the skew of maximum size distribution (Fig. 3), although there was an observable pattern in dunes 5–17. However, early in dune succession, the environment may be too unstable for competition to be a major factor affecting community assembly, as “potential physical stresses related to sand movement, wind, and evaporation decrease…with increasing dune age” (Lichter 1998 p. 500) at this study site. The earliest dune that may be considered stable is dune 5, based on the presence of established tree species, and sand aggregation (refer to the study site description). It appears, then, that the transition from a disturbance-dominated environment to a competition-dominated environment would occur around dune 5, and that the influence of competition should thus be evident primarily on dunes older than dune 5.

Taking the stability and productivity of the dunes in the chronosequence into account, the skewness coefficient decreases when only dunes 5–17 are considered (Fig. 3). Hence, over time, the relative number of large species increases, as would be predicted if competition becomes more important through succession and if size is an important trait defining competitive ability. While there is little literature on changes in species size distributional skew during succession, the relative increase in large species coincides with the general observation of increasing plant stature along resource gradients (Grime 1979; Tilman 1986), which is thought to reflect the importance of height in competition for light (Givnish 1982).

Despite this negative relationship between plant size skewness and dune number (on the stable dunes), the skewness coefficient on all dunes is nevertheless positive, indicating that species size distributions are right-skewed, and thus, small species are always relatively more abundant than larger species, regardless of dune age. How or why, then, do relatively small species continue to persist if, as traditional theory suggests (e.g., Goldberg 1996), they are competitively inferior? We predicted that if size is the principle determinant of competitive ability, then on older dunes where competition has had time to have an impact on community assembly, large species should exclude small species at least at the local neighborhood scale, thus causing near neighbors to be similar in size. In contrast, the ASD between interspecific neighbors based on maximum recorded size (i.e., maximum ASD) was significantly greater that expected by chance in seven of the 17 dunes, with no clear temporal trend (Table 1). Four of the seven dunes with significant maximum ASDs make up the early part of the chronosequence in which disturbance and stress factors are expected to be more important than competition. Moreover, only three of the 17 dunes had an ASD less than 0.5000. This suggests that there is very little tendency for interspecific neighbors to be similar in size—contrary to what might be expected if local competitive exclusion was occurring based on size-asymmetric competition.

The disparate sizes of interspecific neighbors may have several explanations. Smaller species may be shade-tolerant and so may thrive despite proximity to larger species. Smaller species likely also have shallower rooting depths and so may avoid some measure of belowground competition with larger species (Whittaker 1969; King and Woodell 1973; Yeaton et al. 1977; but see also Schurr et al. 2004; Stubbs and Wilson 2004). These issues relate to the potential for “packing constraints” on how close individuals of certain species may be based on their sizes. Larger species, by their sheer size, require a large “physical-space-niche” (Aarssen et al. 2006). Because of inefficiency of resource use associated with increasing plant size, larger species leave more resources unused within their physical-space-niche; unused resources will be present as small patches (e.g., light gaps) (e.g., Beatty 1984; Breshears et al. 1997). These patches are too small for another large individual, but provide enough resources and space to allow a smaller species to reach reproductive maturity.

All of the species sampled on the dune chronosequence are perennials; hence, sampled individuals were at various life stages, and subsequently were different in actual size. As competitive interactions are known to change during the life span of some perennials (e.g., Rejmanek and Leps 1996), it may be more appropriate to consider the difference in actual measured size of the individuals, as this may better reflect the current interaction dynamics. Similarly, the use of only interspecific neighbors may limit the ability to capture the consequences of interactions among plants. The influence of neighbors on a target plant decreases with distance from the target plant (Weiner 1984); interspecific neighbors on the dunes are not necessarily the nearest neighbors for some individuals and therefore, may not be the individual with which a point species is interacting most directly.

ASDs based on measured sizes of nearest neighbors reveal that neighbors on nearly all of the dunes are significantly similar in measured size (Table 1), consistent with the size-advantage hypothesis of competitive ability. This would suggest that neighborhood competition may have been important initially, and now species that coexist at a very local scale are competitively equivalent. However, the use of nearest neighbors does include the potential for intraspecific neighbors. Since intraspecific neighbors have a predisposition to being similar in size on a particular dune, and there is a propensity for nearest neighbors to be intraspecific (especially for clonal species), the use of measured size of nearest neighbors may be too liberal a test to infer the influence of size on spatial patterns. Roughly half of the species recorded have some form of clonal propagation (based on descriptions in Gleason and Cronquist (1991)) but there was no temporal pattern of clonal species frequency across the dune chronosequence (i.e., younger dunes were just as likely as older dunes to include clonal resident species).

When the potential for intraspecific neighbors is removed by using interspecific neighbors only, while maintaining the measured size of plants for calculating the ASD, nearly all dunes (14 out of 17) had nonsignificant ASDs (Table 1). Thus, neighboring species are neither more similar nor more different in measured size than expected by chance. This suggests that the inherent similarity in size of intraspecific neighbors influenced the result of the nearest-neighbor analysis. Furthermore, it suggests that species size is not, or has not been, a predominant factor influencing interspecific spatial pattern on these dunes, which is consistent with the hypothesis that body size is not the only—or even the primary—trait defining between-species competitive ability in plants. This parallels results of a recent study (Keating and Aarssen 2009) showing that larger species in woody vegetation do not generally limit the number of species that can coexist within their immediate neighborhoods. Schamp et al. (2008) also found that species co-occurrence in old field plots was generally random and unrelated to species height, while Turnbull et al. (2004) found that co-occurrence decreased with increasing biomass dissimilarity of desert annuals. It is difficult to fully compare the results of this study with that of Schamp et al. (2008) and Turnbull et al. (2004) because these two studies analyzed co-occurrence of species pairs in plots that contained many other potential competitors, which may or may not affect the interaction dynamics between any given species pair. Research using contact sampling techniques (de Jong et al. 1983) or point sampling would be most appropriate for comparison to the current study. The only study that analyses height similarity/dissimilarity of coexisting species at the point scale is Stubbs and Wilson's (2004) investigation of a sand dune community. In their analysis, species height does not appear to show any tendency toward either limiting similarity or character divergence, which is consistent with the current study.

The dispersion analysis for seed size also failed to support the size-advantage hypothesis for competitive ability—that is, neighboring species did not have similar seed sizes. In fact, neighboring species tended to have different seed sizes: more than half of the dunes have significantly larger ASDs for seed size than expected by chance (Table 1). As with the maximum size ASDs, almost all of the probabilities for seed size ASDs for the chronosequence are greater than 0.5000, generally indicating a tendency for disparate seed sizes between near neighboring species. These results are contrary to the data reported by Turnbull et al. (2004) showing evidence that co-occurring species (at the quadrat scale) were more similar in seed size, and that negative associations were more common among species with different seed sizes. Similarly, Epp and Aarssen (1989) found that co-occurring grassland species tended to have similar seed mass.

The disparity in seed size of neighboring species may simply be related to the same disparity in maximum height of neighbors (or vice versa). That is, plant size and seed size are positively correlated (Leishman et al. 1995), so the finding that one characteristic has a particular spatial pattern may not be independent of the other characteristic.

## Conclusions

We found that across 485 years of sand dune succession, nearest-neighboring pairs of species were generally no different from each other in their relative body sizes than expected from a random assembly of resident species (Fig. 1). For several dunes, seed sizes of neighboring species were more different than expected by random assembly. These results suggest either that (1) competition is not particularly important in affecting the assembly of these dune communities—that is, facilitation may be equally important, with counteracting effects (Brooker et al. 2008)—or, (2) that relative size is not all that matters in defining relative competitive ability (i.e. the ability to recruit offspring, under crowded conditions, into future generations). We suggest that the latter interpretation is particularly in need of further study. The persistent spatial randomness observed throughout the sand dune chronosequence, for trait dispersion (this study) as well as for interspecific associations (Waugh and Aarssen 2011), is consistent with non-niche theories of community assembly based on competitive equivalence (Aarssen 1983, 1989, 1992, 2005; Hubbell 1997, 2005, 2006). The ability to produce descendants despite intense competition will depend on more than just plant body size or seed size; the ability to survive (e.g., through shade tolerance) and produce offspring (e.g., through reproductive economy), despite intense competition, should be at least equally important, yet few studies have measured all three of these components of competitive ability (Aarssen & Keogh 2002; Aarssen 2005). Studies of the interaction and relationships among these three components, we suggest, are essential for future advances in our understanding of the mechanisms of plant community assembly.
